# Alveolar rhabdomyosarcoma of cervix: A case report

**DOI:** 10.1097/MD.0000000000043675

**Published:** 2025-08-01

**Authors:** Xiuli Zhang, Junfen Xu

**Affiliations:** a Department of Gynecologic Oncology, Women’s Hospital, Zhejiang University School of Medicine, Hangzhou, Zhejiang, China.

**Keywords:** alveolar rhabdomyosarcoma, case report, cervix, chemotherapy, surgery, treatment

## Abstract

**Rationale:**

Alveolar rhabdomyosarcoma (ARMS) is a rare and highly aggressive malignant soft tissue tumor. ARMS is associated with a poor prognosis, especially in adults, occurring in the uterine cervix infrequently. To date, only 6 cases have been reported in the literature. We discuss the challenges in diagnosing and managing cervical ARMS and highlights the need for ongoing research into optimal treatment strategies for this malignancy.

**Patient concerns:**

A 51-year-old female was diagnosed with advanced-stage ARMS. Positron emission tomography and computed tomography scans indicated a high metabolism mass in the uterus region, along with multiple lymph nodes enlargement in the pelvic, para-aorta and mediastinal regions. Cervical biopsy and segmental curettage revealed a small cell malignant tumor with poor differentiation. Histological and immunohistochemical examination confirmed ARMS.

**Diagnoses:**

The final diagnosis was cervical ARMS, staged IV according to the Intergroup Rhabdomyosarcoma Studies Group based on the imaging and histology results.

**Interventions:**

The patient underwent radical hysterectomy, bilateral salpingo-oophorectomy, pelvic lymphadenectomy, and para-aortic lymphadenectomy. Postoperatively, the patient received a series of chemotherapy regimens including VAC^†^ (vincristine, epirubicin, cyclophosphamide), EP (etoposide and cisplatin), VIP (etoposide, ifosfamide, cisplatin), gemcitabine and bevacizumab, gemcitabine and docetaxel, pertuzumab and lenvatinib and radiotherapy.

**Conclusion:**

ARMS has low incidence with unique clinical pathological characteristics. The biological behavior is more aggressive and the prognosis is worse in ARMS. Further research is necessary to refine treatment protocols and improve survival rates for this aggressive tumor.

## 1. Introduction

Rhabdomyosarcoma (RMS) is a malignant soft tissue sarcoma that arises from embryonic mesenchymal stem cells and is characterized by rhabdomyoblasts.^[1]^ It is classified into 4 subtypes: embryonal, alveolar, pleomorphic and botryoid.^[2]^ Alveolar rhabdomyosarcoma (ARMS) amounts for 20% to 30% of all RMS cases.^[3]^ The occurrence of ARMS in the uterine cervix is exceedingly rare, with only 6 cases documented in the literature to date. The first case was reported by Emerich et al in 1996, the patient was diagnosed at an advanced stage, underwent surgery followed by radiotherapy but succumbed to the disease within 3 months.^[4]^ Similar cases of advanced-stage ARMS had poor outcomes despite aggressive treatments, including surgery, chemotherapy, and radiotherapy.^[5,6]^ Conversely, early-diagnosed cases have shown a more favorable prognosis.^[7–9]^

Here, we present a case of cervical ARMS in an adult woman diagnosed at a late stage, and discuss the treatment options available for this rare and aggressive malignancy.

## 2. Case report

**Figure 1.**
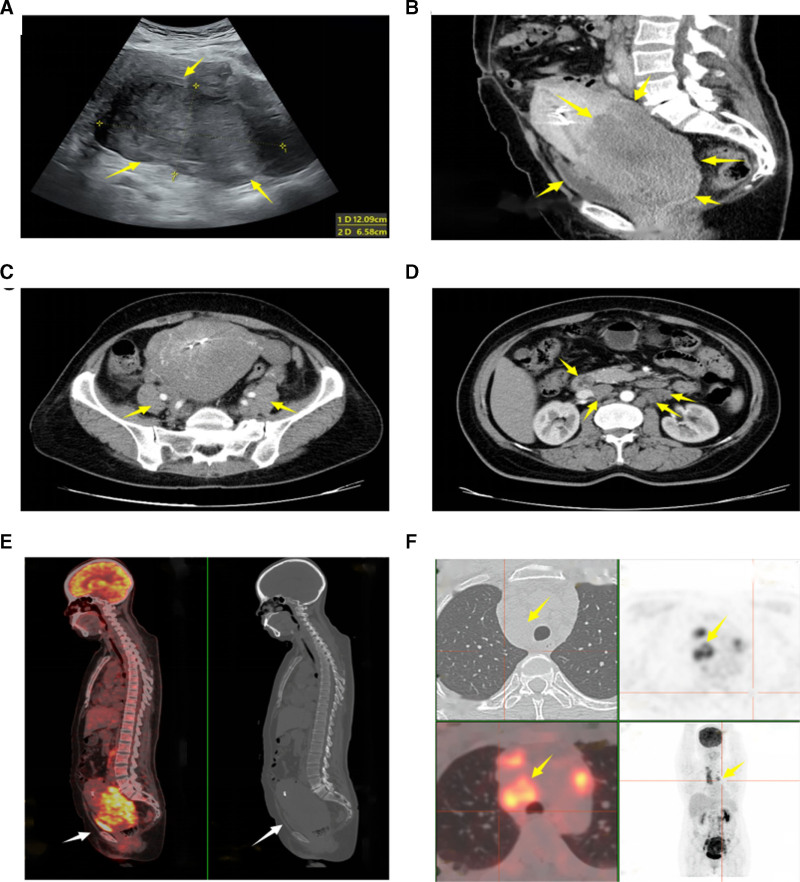
Imaging indicated the mass and lymph nodes. (A) Ultrasound showed a hypoechoic lesion extending from the anterior uterine wall to the cervix. (B–D) Computed tomography scan showed enlarged: cervix, uterus (B), pelvic lymph nodes (C) and para-aortic lymph nodes (D). (E and F) PET/CT demonstrated the mass in the pelvic cavity (E) and metastasis of mediastinal lymph nodes (F). CT = computed tomography, PET = positron emission tomography.

A 51-year-old postmenopausal woman presented with a pelvic mass persisting for 7 months. On physical examination, a large, firm, hypertrophic, and fixed pedunculated mass was found protruding from the cervix. The mass in the cervix measured approximately 6-cm and caused bilateral paracentral adipose space loss. Ultrasound imaging demonstrated a hypoechoic lesion measuring 12.1 × 9.3 × 6.6 cm, extending from the anterior uterine wall to the cervix (Fig. 1A). CT identified a low-density mass of 8.0 × 13.4 × 8.7 cm within the cervix, along with enlarged pelvic and abdominal aortic lymph nodes, the largest measuring 2.0 cm (Fig. 1B and D). Additionally, 18-FDG PET-CT demonstrated lymph node enlargement in the pelvic, para-aorta, and mediastinal regions (Fig. 1E and F). The patient had a slightly elevated serum CA125 (59 U/mL, normal range: <25.0 U/mL) and CA199 (50.7 U/mL, normal range: <30.0 U/mL). Both pap smear and human papillomavirus (HPV) tests were negative.

**Figure 2.**
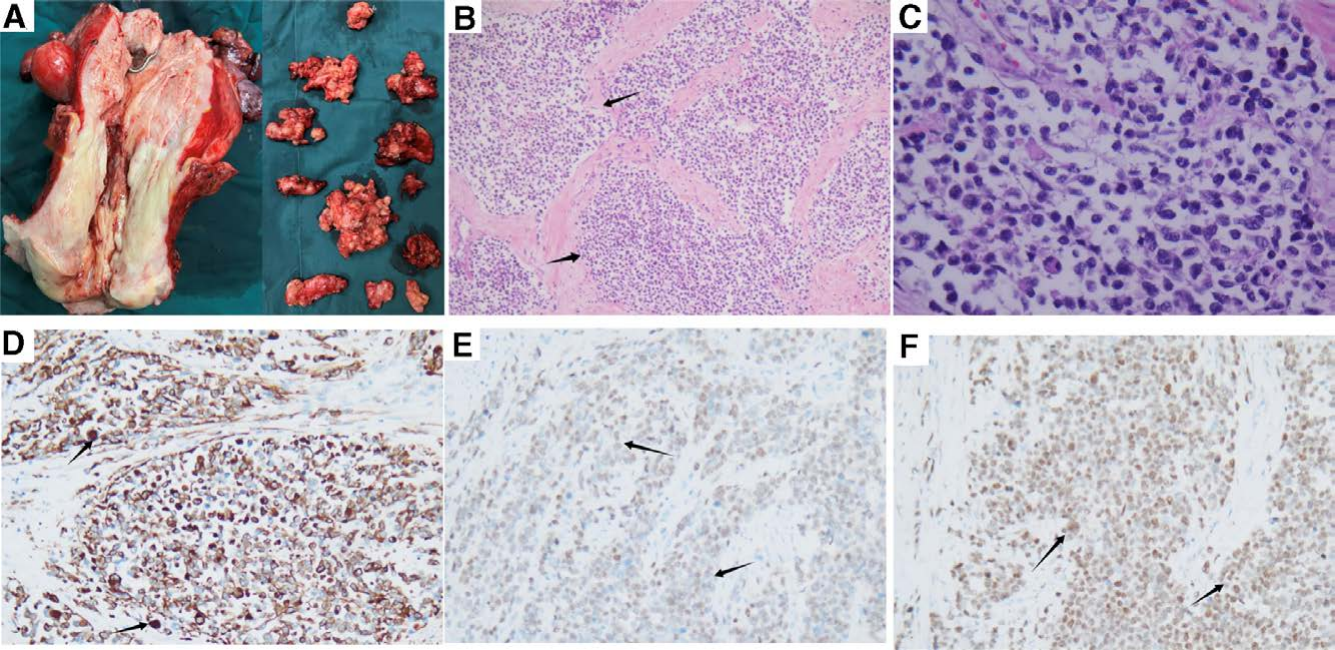
The characteristics of pathology IHC. (A) Gross image showing tumor in in the cervix and uterus, abnormally enlarged lymph nodes. (B) Low-power image reveals a nested alveolar pattern of tumor cells, divided by fibrous septa. (C) High-power image displaying a solid variant with sheets of medium-sized tumor cells. (D–F) Immunohistochemical stain of the tumor: desmin is positive in smooth muscle cytoplasm (D), MyoD1 positivity in smooth muscle nucleus(arrows) (E), Myogenin was diffusely and strongly positive in the nuclear of the tumor cells (F). IHC = immunohistochemistry.

Cervical biopsy and segmental curettage revealed a small cell malignant tumor with poor differentiation. After a multidisciplinary discussion, the patient underwent radical hysterectomy, bilateral salpingo-oophorectomy, pelvic lymphadenectomy and para-aortic lymphadenectomy. The gross specimen showed a solid grayish tumor extending through the cervix and uterus, both ovaries and multiple lymph nodes were involved by the tumor (Fig. 2A).

**Figure 3.**
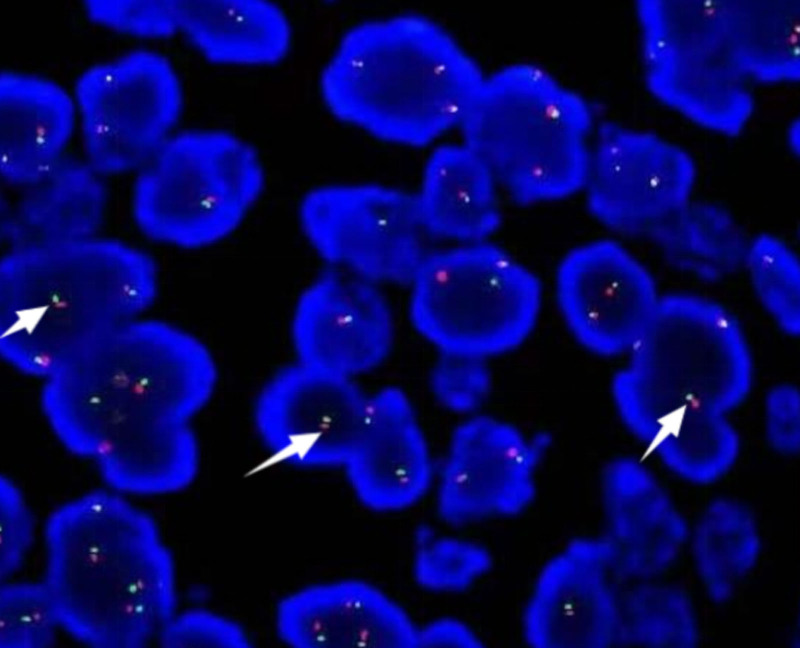
Fluorescence in situ hybridization: dual-color, break-apart interphase FISH split red and green signals indicate the presence of an FOXO1(13q14) gene rearrangement involving 1 chromosome, the fused signals indicated an intact 13q14 allele (arrows). FISh = fluorescence in situ hybridization.

Histologically, these highly cellular neoplasms consist of primitive cells with round nuclei, divided by fibro-vascular septa into distinct nests. These nests exhibit central cell clusters with peripheral cohesion loss, creating an ‘alveolar’ appearance (Fig. 2B and C). Immunohistochemically, ARMS stain showed that the tumor was strongly positive for Desmin, MyoD1, and Myogenin (Fig. 2D–F). The negativity of the tumor for cytokeratin ruled out an epithelial tumor. Negative P16 and HPV detection indicated that this cervical malignancy was not associated with HPV infection. However, fluorescence in situ hybridization (FISH) studies for FOXO1 fusion were negative, polyploid changes were observed in 70% of the cells (Fig. 3). According to the International Classification of Rhabdomyosarcoma and World Health Organization guidelines, the differential diagnosis of alveolar rhabdomyosarcoma (ARMS), excluding the solid variant, necessitates the identification of histopathological regions characterized by distinctive alveolar space.^[10]^ Histologically, the neoplasms are highly cellular and consisting of primitive cells with round nuclei. Fibro-vascular septa divides the tumor into distinct nests. It has a feature of central cell clusters and peripheral cohesion loss, making it has an “alveolar” look. The pattern of the histology and the immunohistochemical staining confirmed the diagnosis of cervical ARMS, staged IV according to the Intergroup Rhabdomyosarcoma Studies Group based on imaging and histology.

Postoperatively, the patient underwent a series of treatments summarized in Table 1. Initially, she received 1 cycle of the VAC^†^ (vincristine, epirubicin, and cyclophosphamide) chemotherapy. However, she developed facial edema and enlargement of the neck and supraclavicular lymph nodes, which required double upper arm venous angiography, stent placement, and balloon dilation. Given disease progression, the chemotherapy regimen was switched to EP (etoposide and cisplatin), followed by 4 cycles of the VIP (etoposide, ifosfamide, and cisplatin) regimen. Despite these interventions, imaging showed further disease progression, leading to the addition of gemcitabine and bevacizumab to the treatment plan. The patient then received chemotherapy with gemcitabine and docetaxel. This was followed by radiotherapy to the mediastinal and bilateral supraclavicular regions, delivering 42 Gy in 14 fractions to 95% of the planned treatment volume. The treatment regimen was subsequently modified to include pertuzumab and lenvatinib. Additional radiotherapy was administered to the pelvic and inguinal regions, with a dose of 30 Gy in 12 fractions. Despite the ongoing treatment, the patient continued to experience disease progression and ultimately passed away 11 months post-diagnosis.

**Table 1:** Treatment timeline.

Date	phase	Treatment	Regimen details	Response
February 22, 2024	Surgery	RH + BSO + SPBL + PALA	Histology, IHC and FISH proved ARMS	Well-healed incision
March 09, 2024	Adjuvant	Chemotherapy (VAC)	Epirubicin 100 mg, vindesine 3 mg, ifosfamide1400 mg iv, q3w	Enlarged lymph nodes compressed the superior vena cava, caused facial edema
April 24, 2024	Invasive procedures	Bilateral brachiocephalic venography	Stent implantation and balloon dilation	Facial edema got remission
April 30, 2024	Adjuvant	Chemotherapy(EP + Anlotinib)	Etoposide150 mg and cisplatin 30 mg × day 1–3 iv, anlotinib 12 mg po qd	Progression of disease
May 23, 2024–August 02, 2024	Adjuvant	Chemotherapy (VIP) × 4	Etoposide150 mg, ifosfamide1400 mg, cisplatin 30 mg × day 1–5 iv	CT scan indicated disease progression
August 28, 2024	Adjuvant	Chemotherapy(gemcitabine + docetaxel)	Gemcitabine1400 mg day 1, day 8, bevacizumab 400 mg day 1, docetaxel 110 mg day 8 iv	Disease progressed, radiotherapy was planed
October 15, 2024	Radiotherapy	Mediastinal and bilateral supraclavicular lymph node metastases keep progressing	95% PTV:42GY/3GY/14F	Well tolerated
October 19, 2024	Adjuvant	Immunotherapy	Putrizumab 200 mg iv, lenvatinib 12 mg po qd	Disease progression still couldn’t be controlled.
November 13, 2024	Radiotherapy	Pelvic and inguinal lymph nodes metastases	95% PTV:30GY/2.5GY/12F	
November 11, 2024	Adjuvant	Targeted therapy	Putrizumab 200 mg iv and lenvatinib 12 mg po qd	Died on January 08, 2025

iv = intravenous infusion, po = per os, PTV = planned treatment volume.

## 3. Discussion

ARMS is an aggressive malignancy, typically found in the extremities and rarely in the cervix. The occurrence of ARMS in the cervix is so uncommon that only 6 cases have been documented in the literature. The patients in these cases were all under 55 years of age, with the present case involving a 51-year-old woman, consistent with the demographics of this rare tumor.

Cervical ARMS usually presents as a rapidly growing, nor-tender mass. It can metastasize early to lymph nodes and distant sites, which complicates treatment and makes the prognosis worse. The most common presenting symptom in adults is vaginal bleeding, though our patient presented with a mass without significant pain. Imaging plays a crucial role in the diagnosis, while biopsy and histopathology are definitive for confirming ARMS.

There are currently no significant tumor markers for ARMS. Genetic analysis using a FISH assay on paraffin-embedded tissue sections can be beneficial for diagnosis and serve as an adjunct to the diagnostic process. FOXO1 fusion is a genetic abnormality commonly associated with 80% of ARMS and a known poor prognostic marker.^[11]^ In this case, although FISH analysis did not detect FOXO1 fusion, histologically showed typical picture of ARMS and the results of immunohistochemistry also points to the diagnosis of ARMS. The literature shows that fusion-positive ARMS correlates with poorer overall survival (OS) and event-free survival.^[12]^ This highlights the importance of molecular diagnostics not only for confirming the diagnosis but also for guiding prognosis and treatment decisions. The final diagnosis confirmed through pathological and immunohistochemical staining results, including Desmin and Myogenin, alongside FISH examination results. As highlighted in the 6 previously reported cases, patients classified as Intergroup Rhabdomyosarcoma Studies Group stage IV experienced rapid deterioration following diagnosis. Our patient had an OS of 11 months after diagnosis. As reported by Gallego et al in the European pediatric Soft tissue sarcoma Study Group (EpSSG) cohort, the 5-year OS of patients with FOXO1 fusion-positive tumors was 51.6%, compared to 76% for those with FOXO1 fusion-negative tumors, which approached statistical significance (*P* = .069).^[12]^

Due to the rarity of cervical ARMS, there are no established treatment guidelines. To gain insight into the management of this rare tumor, we reviewed the 6 previously reported cases of cervical ARMS^[4–9]^ and summarized the treatment strategies in Table 2. Notably, all cases, except for one that only underwent surgery and showed no evidence of metastasis, received some forms of chemotherapy. Three patients were treated with the VAC (cyclophosphamide, actinomycin, vincristin) regimen, which is consistent with the standard treatment of RMS, a tumor that typically responds well to chemotherapy. This regimen is widely used to improve survival rates and minimize the risk of recurrence, and 2 of these patients did not experience recurrence.^[13,14]^ Another patient received a VAI (vincristin, actinomycin-D, ifosofamide) regimen, however, this patient succumbed to the disease within 3 months. While some studies suggest the potential benefit of adjuvant combination chemotherapy, its efficacy remains inconclusive, as noted by Ashley et al.^[5]^ In our case, despite undergoing multiple chemotherapy regimens, including VAC^†^(vincristine, epirubicin, cyclophosphamide), EP (etoposide and cisplatin), VIP (etoposide, ifosfamide, cisplatin), as well as radiotherapy and targeted therapies such as pertuzumab and lenvatinib, the patient’s disease continued to progress. This further highlights the limited understanding of effective treatment strategies for cervical ARMS, underscoring the need for more robust clinical data. Although the optimal treatment remains uncertain, the experience from these cases offers hope for future advancements in patient care and quality of life.

**Table 2:** Cases of alveolar rhabdomyosarcoma of cervix reported in the literature.

Case	Age (year)	Clinical presentation	Size (cm)	IRSG	FOXO1 fusion	Treatment	Metastasis	Clinical outcome
Present case (case 1)	51	Pelvic mass	13.4	IV	Negative	RH + BSO + SPBL + PALA Systemic chemotherapy VAC^†^, EP, VIP, immunotherapy, antiangiogenic therapy, radiotherapy	Lymph node	Died (11 mo after surgery, died from extensive tumor metastasis)
Janusze Merich et al (case 2)	45	Vaginal bleeding difficulties with micturition Weight loss	15	IV	Unknown	TAH + SPBL EBRT intracavitary brachytherapy	Lymph node	Died (3 mo after surgery died from fulminant disseminated neoplastic disease)
Ng et al (case 3)	39	Vaginal bleeding	6	I	Unknown	TAH + LSO + SPBL + PALA Systemic chemotherapy* 3 cycles Pelvic irradiation	No metastasis	NED 36 mo
Case et al (case 4)	21	Excessive vaginal bleeding	10.2	I	Unknown	NACT:VAC 5 cycles TAH + BSO VAC 5 cycles	No metastasis	NED 20 mo
Rivasi et al (case 5)	49	Vaginal bleeding	4	I	positive	TAH + BSO	No metastasis	NED 18 mo
Odoi et al (case 6)	44	Abdominal mass Vaginal bleeding Weight loss	26 wk gravid uterine size	IV	positive	Uterine and omental biopsies	Omental peritoneal, bowel and anterior abdominal wall	Died
Cakar et al. (case 7)	18	Vaginal bleeding Mass protruding from vaginal	11	IV	Unknown	TAH + SPBL + PALA VAI 5 cycles Radiotherapy	Right obturator space	Died (3 mo later, huge intraabdominal mass, massive ascites)

BSO = bilateral salpingo-oophorectomy, EBRT = external beam radiotherapy, EP = etoposide, cisplatin, FIGO = International Federation of Gynecology and Obstetrics, IHC = immunohistochemistry, IRSG = Intergroup Rhabdomyosarcoma Studies Group, LSO = left salpingo-oophorectomy, mo = months, NACT = neoadjuvant chemotherapy, PALA = para-aortic lymphadenectomy, RH = radical hysterectomy, SPBL = systematic pelvic bilateral lymphadenectomy, TAH = total abdominal hysterectomy, VAC = cyclophosphamide, actinomycin, vincristin, VAC^†^ = vincristine, epirubicin, cyclophosphamide, VAI = vincristin, actinomycin-D, ifosofamide, VIP = etoposide, ifosfamide, cisplatin.

* Cyclophosphamide, epirubicin, etoposide, ifosfamide, mesna.

## 4. Conclusions

Cervical ARMS is an aggressive malignancy that presents significant challenges in diagnosis and management. Due to its rarity, there is no consensus on the optimal treatment approach. However, this case and the review of the literature emphasize the importance of early recognition, multidisciplinary treatment strategies, and the potential role of molecular diagnostics in improving patient outcomes. Further research is necessary to refine treatment protocols and improve survival rates for this rare and aggressive tumor.

## Author contributions

**Conceptualization:** Junfen Xu.

**Funding acquisition:** Junfen Xu.

**Writing – original draft:** Xiuli Zhang.

**Writing – review & editing:** Junfen Xu.
